# A review of recent publication trends from top publishing countries

**DOI:** 10.1186/s13643-018-0819-1

**Published:** 2018-09-27

**Authors:** Paul Fontelo, Fang Liu

**Affiliations:** National Library of Medicine, 8600 Rockville Pike, Bethesda, MD 20894 USA

**Keywords:** PubMed, Publication type, Meta-analysis, Systematic review, Collaborative publishing, Evidence-based medicine

## Abstract

**Background:**

Evidence-based medicine relies on current best evidence from the medical literature, the patient’s history, and the clinician’s own experience to provide the best care for patients. Systematic reviews and meta-analysis are considered the highest levels of evidence for informing clinical decisions. Recently, reports have shown an increase in the number but a decrease in quality of meta-analysis publications. We reviewed publication trends and determined the countries with the most journal articles and types of publications in PubMed from 1995 to 2015.

**Methods:**

We examined journal entries in PubMed from 1995 to 2015 from top publishing countries for total number of publications and citations in core clinical journals and in specific publication types (systematic reviews, meta-analysis, randomized controlled trials).

**Results:**

Yearly, only 30 countries generated 94.6% of all publications and 98.1% of core clinical journals worldwide. All publication types increased but with a significant increase in meta-analysis publications from China. Collaborative and co-authored papers among the 30 countries also showed an increasing trend.

**Conclusion:**

The USA leads in all publication citations and specific publication types, except for meta-analysis where China publishes more. Collaborative publishing among international collaborators is also increasing.

## Background

Recent best evidence from the literature and health care providers experience form the foundation of evidence-based medicine (EBM) [1]. In the hierarchy of evidence, randomized controlled trials, systematic reviews, and meta-analysis studies are at the highest levels in the evidence pyramid [2]. Meta-analysis and review articles are often the most highly cited publications [3]. Recently, the exponential increase in the number of meta-analysis studies has raised the issue of quality and reliability of meta-analysis publications [4–7]. The implications could be consequential because of the high value assigned to meta-analysis and systematic review studies in informing clinical decisions.

The publication type category in the MeSH translation table represents the article’s type of material (ex: case report, clinical trial, editorial, guideline, meta-analysis, etc.) [8]. The publication type in PubMed represents an article’s “study design” and not the “type of material.”

This paper is an attempt to review recent trends of some publication types in PubMed. It is not an assessment of the quality of journal articles themselves but rather a quantitative look at recent publication trends from top research countries worldwide.

## Methods

PubMed searches were performed in December 2016 using the following limits: all publications sorted from top publishing countries from 1995 to 2015 and the following search strategies and publication types: meta-analysis, systematic reviews, clinical trials, and randomized controlled trials. A list of countries was created (*n* = 235) based on IPv4 Internet address allocations (https://en.wikipedia.org/wiki/List_of_countries_by_IPv4_address_allocation). The top publishing countries was generated from searches of all publications in PubMed for the year 2015. An example of a country search query for Germany is in Appendix 1. The number of publications in 2015 for each country was sorted, and the top 25 countries with the most publications were selected. Although the limit was initially set to the top 25 countries based on the total number of publications, five other countries that were not on the original list were found to be in the top 25 when specific publication types were analyzed. This brought the total highest publishing countries to 30 countries and regions. All journal articles from the 30 countries published between 1995 and 2015 in journals listed in National Library of Medicine (NLM)’s Abridged Index Medicus (AIM) or “Core Clinical” Journal Titles subset were also searched for publication types labeled as meta-analysis, systematic review, clinical trial, and randomized controlled trial. Examples of search filters for AIM journals are shown in Appendices 2, 3, and 4. The AIM or “Core Clinical” is a subset of MEDLINE [8] which currently includes about 119 journals. Publications from the 30 countries were reviewed in AIM subset [9, 10]. Collaborative or co-authored publications were determined by searching for citations with combined affiliations between each of the top 5 countries and the 30 other countries on the list. In this paper, the term “citation” refers to the entry record of a publication in PubMed, not in its use in the reference section of a manuscript.

An expansion process for a country’s name filter was performed to include the common expression in the affiliation field because of recent changes in PubMed (https://www.nlm.nih.gov/pubs/techbull/so13/brief/so13_author_affiliations.html). For example, both “UK” and “United Kingdom” and “Russia” and “Russian Federation” were included in the affiliation filter to search for publications from the UK and Russia, respectively. Changes in indexing of affiliation information were instituted by the NLM in late December 2013 that required a modification of the search algorithm (https://www.nlm.nih.gov/pubs/techbull/so13/brief/so13_author_affiliations.html). For the USA, the search algorithm was modified by adding all the names of the 50 states in the USA to compensate for this change in practice. Filter algorithms were continuously revised when “unexpected” results were encountered, such as, when entries from New Mexico state were included in results from Mexico, the country (Appendix 5).

A summary of search procedures for generating the data is as follows: Step 1: Find the countries with the most publications. Filters used in this step were affiliation filter and publication date filter. Step 2: Search for selected publication types from top 30 countries. These searches consisted of three filters: affiliation filter, publication date filter, and publication type filter. Step 3: Search for publications from top countries with selected publication types in core journals. Four search filters were used for this step: affiliation filter, publication date filter, publication type filter, and core journals filter. Step 4: Finally, find collaborative publications from the top publishing countries. Results were analyzed using Microsoft Excel. The search algorithms used in this study are shown in Appendices 1, 2, 3, 4, and 5.

## Results

**Fig. 1:**
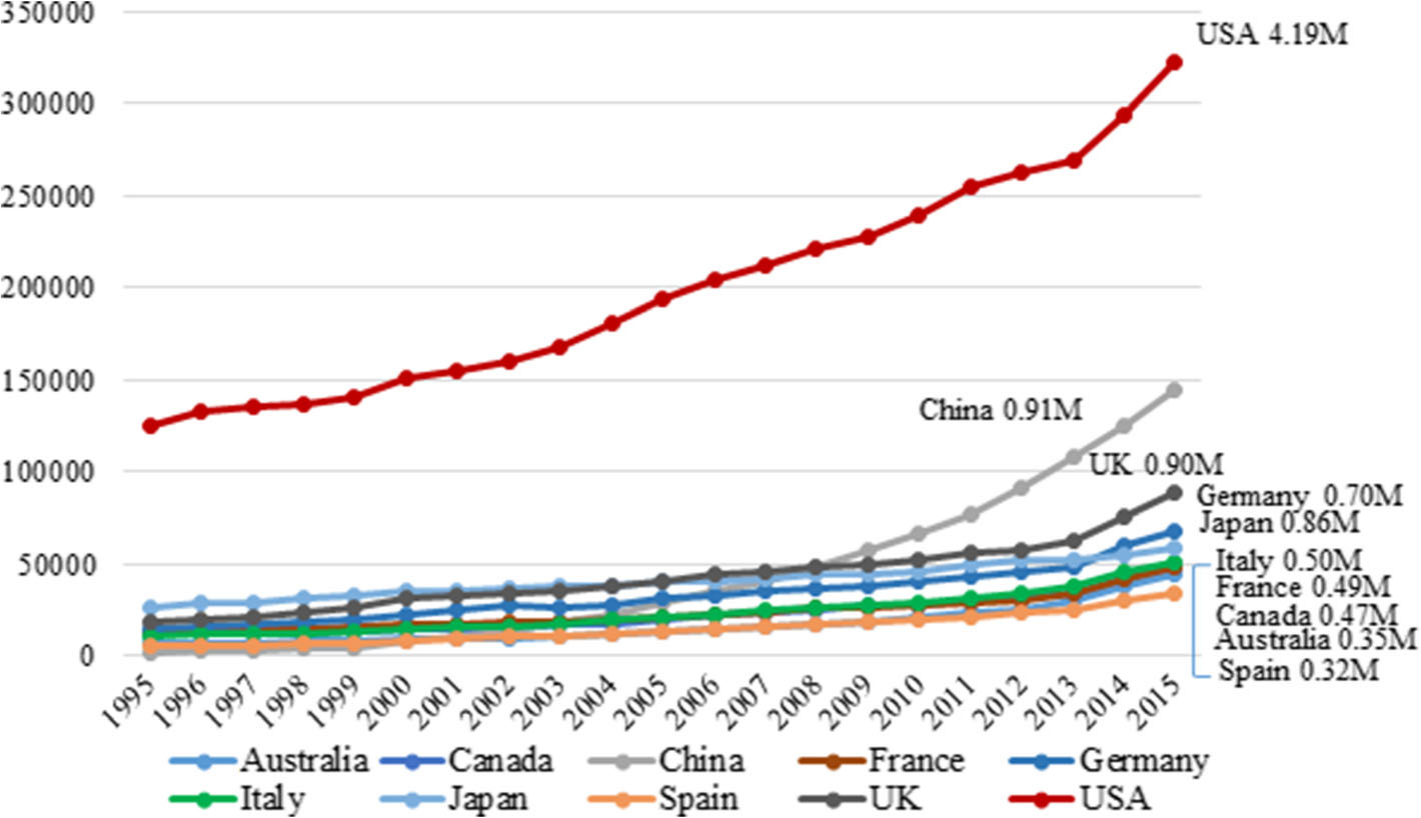
The top 10 publishing countries and total number of entries in PubMed from 1995 to 2015

All data were from PubMed entries from January 1995 to December 2016. In Fig. 1, the total number of citations for the top 10 publishing countries from 1995 to 2015 are shown. The other 20 countries are not displayed because the lines were indistinguishable from each other. Research publications from the United States (USA) showed a steady rise and a doubling of publications in the 20-year review period. Starting around 2009, journal articles from China showed an increasing trend that parallels with the USA. Publications from other top publishing countries also showed an increasing trend but at a more gradual pace.

Table 1 shows data for all publications and the different publication types (systematic review, meta-analysis, clinical trials, and randomized clinical trial) from the top 30 publishing countries for 2015. The USA led in all publications, systematic reviews, clinical trials, and randomized clinical trials, but China published the most number of meta-analysis publications with the USA in second place.

**Table 1:**
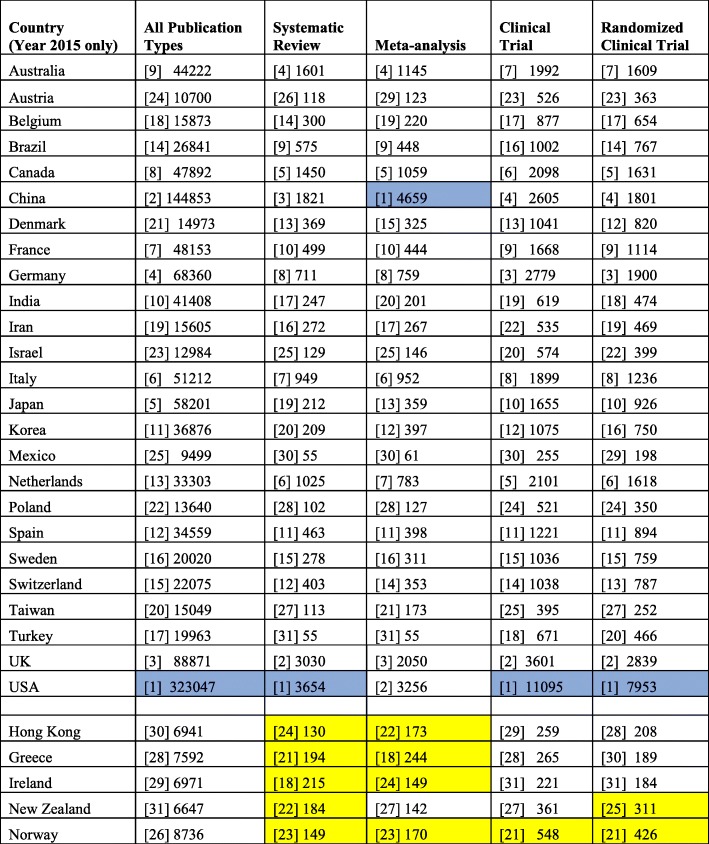
Year 2015 country totals for all publications, systematic review, meta-analysis, clinical trials, and randomized clinical trials. Numbers inside “[]” indicate country rankings for each category. Countries in the last five rows (alphabetically arranged) were not in the original top 25 most published countries but are in the top 25 in publication types (yellow box). Blue box indicates countries with most publications in the category.

Although we originally planned to determine the top 25 highest publishing countries only, we found that there were five other countries and regions that were represented in the top 25 when data for specific publication types were analyzed. This displaced some countries in the original list of top 25 countries in “All publication types.” We subsequently decided to increase the total highest publishing countries and regions to 30 (Table 1). For systematic reviews, five other countries who were in the top 25 were Ireland, 18th; Greece, 21st; New Zealand, 22th; Norway, 23nd; and Hong Kong, 24th. Four countries who were not on the original 25 list but were in the top 25 for meta-analysis publications were Greece, 18th; Hong Kong, 22nd; Norway, 23rd; and Ireland, 24th. Norway was 21st in clinical trial publications and in randomized clinical trials, while New Zealand was in 25th place for randomized clinical trials.

For all publication types in 2015, the USA had more than twice as many publications as China (323,047 vs 144,850), which was second highest (Table 1). The United Kingdom (UK) was third highest at 88,271 or 27.3% of the US total. In 2015, the top 10 countries were (from highest to lowest) US, China, UK, Germany, Japan, Italy, France, Canada, Australia, and Spain.

The Abridged Index Medicus (AIM) or “Core Clinical,” a subset of MEDLINE, [9] which currently lists about 119 journals, contains some of the most highly read and highly cited clinical journals [10]. We reviewed the publications from the 30 countries in AIM subset and found that from 1995 to 2015 (Table 2), the average yearly output from the 30 top producing countries was 93.9% for all publication types and 98.1% for core journals (total number of countries = 235). The average varied only slightly from year-to-year in the 20-year review period.

**Table 2:** Total publications from 30 countries compared to all countries (*n* = 235). Yearly average was 93.9% and 98.1% for all publication types and core clinical journals

Year	All Pub types			Core journals		
	All countries (*n* = 235)	Top 30 only	% 30/235	All countries (*n* = 235)	Top 30 only	% 30/235
1995	291,806	277,457	95.1	30,998	30,110	97.1
1996	313,995	298,289	95.0	33,112	32,197	97.2
1997	324,191	307,440	94.8	34,259	33,267	97.1
1998	341,228	322,652	94.6	34,814	33,839	97.2
1999	358,148	338,015	94.4	35,907	34,818	97.0
2000	399,072	375,098	94.0	36,334	35,287	97.1
2001	421,876	396,344	93.9	36,359	35,383	97.3
2002	444,372	417,469	93.9	37,612	36,647	97.4
2003	473,301	443,748	93.8	38,963	37,906	97.3
2004	514,557	482,641	93.8	40,206	38,710	96.3
2005	570,137	534,627	93.8	41,012	39,248	95.7
2006	614,911	576,849	93.8	40,137	38,987	97.1
2007	655,660	614,277	93.7	40,446	39,338	97.3
2008	702,786	657,358	93.5	40,669	39,593	97.4
2009	741,151	691,626	93.3	40,946	39,829	97.3
2010	799,670	744,345	93.1	41,423	40,277	97.2
2011	872,932	811,309	92.9	42,351	41,317	97.6
2012	937,430	870,338	92.8	41,330	40,282	97.5
2013	977,245	909,156	93.0	41,529	40,724	98.1
2014	1,006,344	945,443	93.9	44,457	43,857	98.7
2015	1,064,266	1,006,618	94.6	48,141	47,245	98.1

**Fig. 2:**
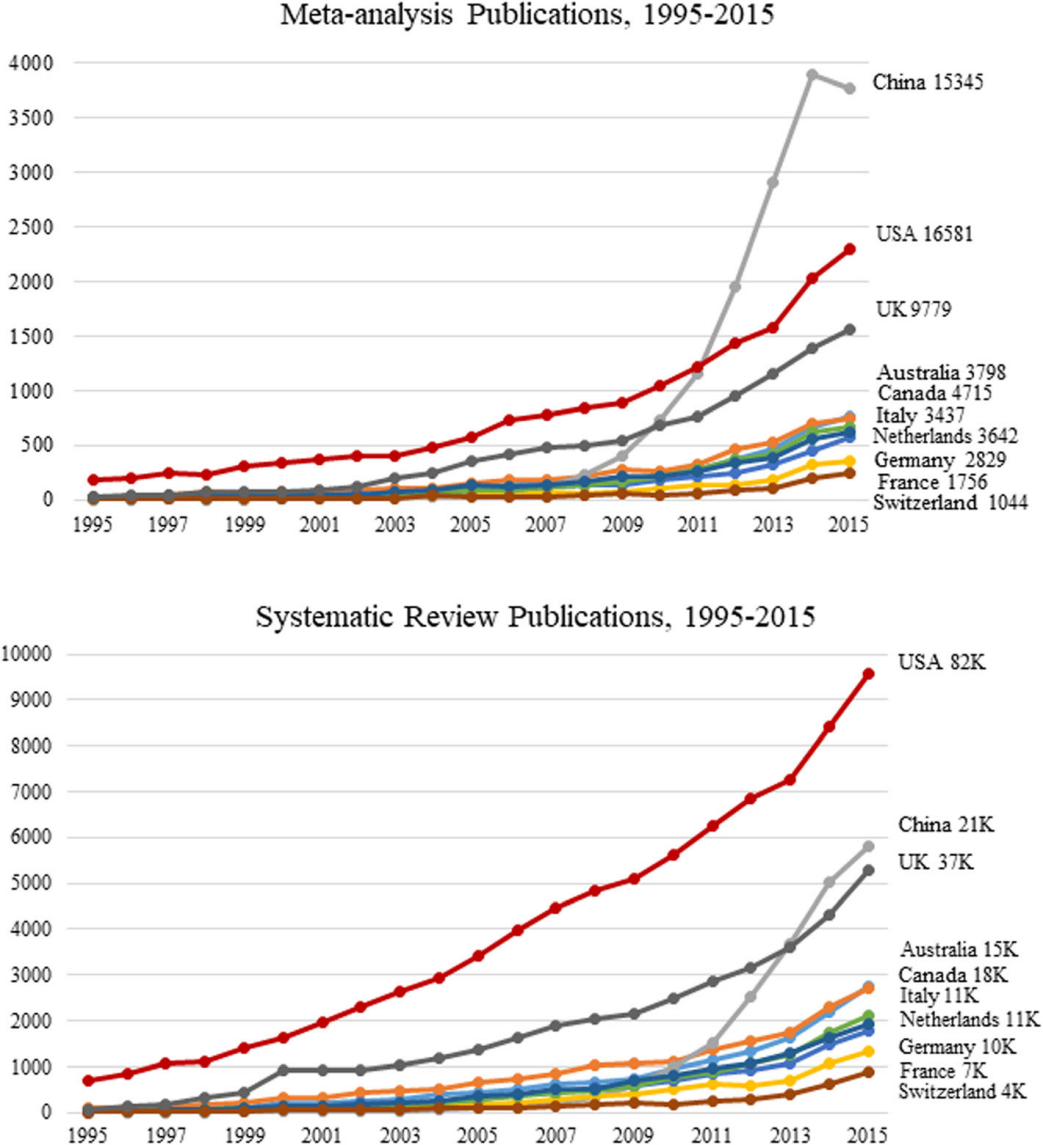
Meta-analysis and systematic reviews all PubMed-indexed publications from 1995 to 2015. Numbers next to the country name indicate 20-year totals

A review of meta-analysis and systematic review publications from the top 10 publishing countries are shown in Fig. 2. The results are similar to that seen in Table 1, where the USA published the most articles in all publications. For China, the rapid growth in meta-analysis publications started in 2009, and eventually surpassing the USA in 2011. Although at the end of 2015 the USA still had more meta-analysis publications than China overall (16,581 vs 15,345), it was already 93% that of the USA and growing at a steeper slope. Compared to the USA, the total percentage of China’s output for total publications, systematic reviews, clinical trials, and randomized controlled trials were much lower—22%, 26%, 14%, and 15%, respectively.

Since “Systematic Review” is not a publication type filter in PubMed, but rather, a subset filter, the search algorithm was modified by including the title and “text word” combination to find articles of that type. The modified filter for systematic reviews was “(systematic review [tw] OR systematic review [tiab] OR meta synthesis [ti] OR cochrane database syst rev [ta] OR systematic literature review [ti] OR pooling project [tw] OR umbrella review [tw])” . Figure 2 shows that the USA leads in systematic reviews that is maintained throughout the 20-year period.

Co-authored publications with the US collaborators account for the highest percentage among the top 30 countries (Table 3). In 2015, the USA published 89,060 papers in collaboration with other countries, which is 27.6% of its total publication output (323,047). Collaborators from China were co-authors in 19.8% (highest) of US total publications, followed by 16.0% from the UK, and 12.9% from Canada. In 2015, China published 34,089 co-authored publications (23.5% of total publications), with 51.7% co-authored with US scientists, 14.4% from Hong Kong, and 9.3% from Taiwan. From 1995 to 2015, there was a mix of decreases and increases in cross country co-authorship; however, the absolute numbers of collaborative publications between the top 5 countries and 30 other countries on the list increased from 2093 in 1995 to 357,746 in 2015. For the other top 4 countries (Table 3), the most co-authored papers were also with the USA. The overall number of co-authored publications for the 30 top research countries, from 1995 to 2015, was 780,521.

**Table 3:**
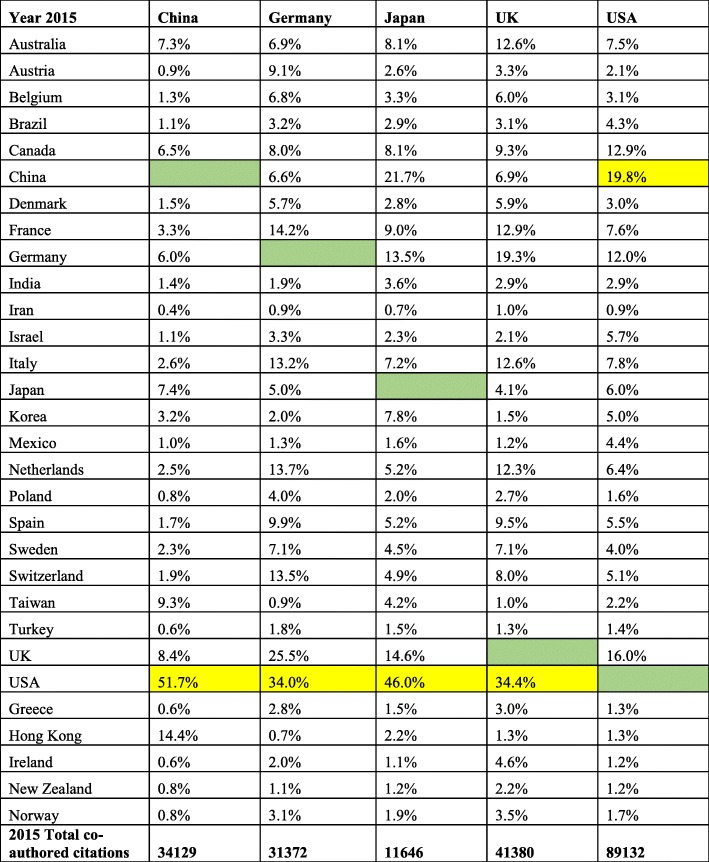
Collaborative publications between the top 5 countries with the 30 other highly published countries for 2015. Yellow boxes indicate the country with the most co-authored papers

**Fig. 3:**
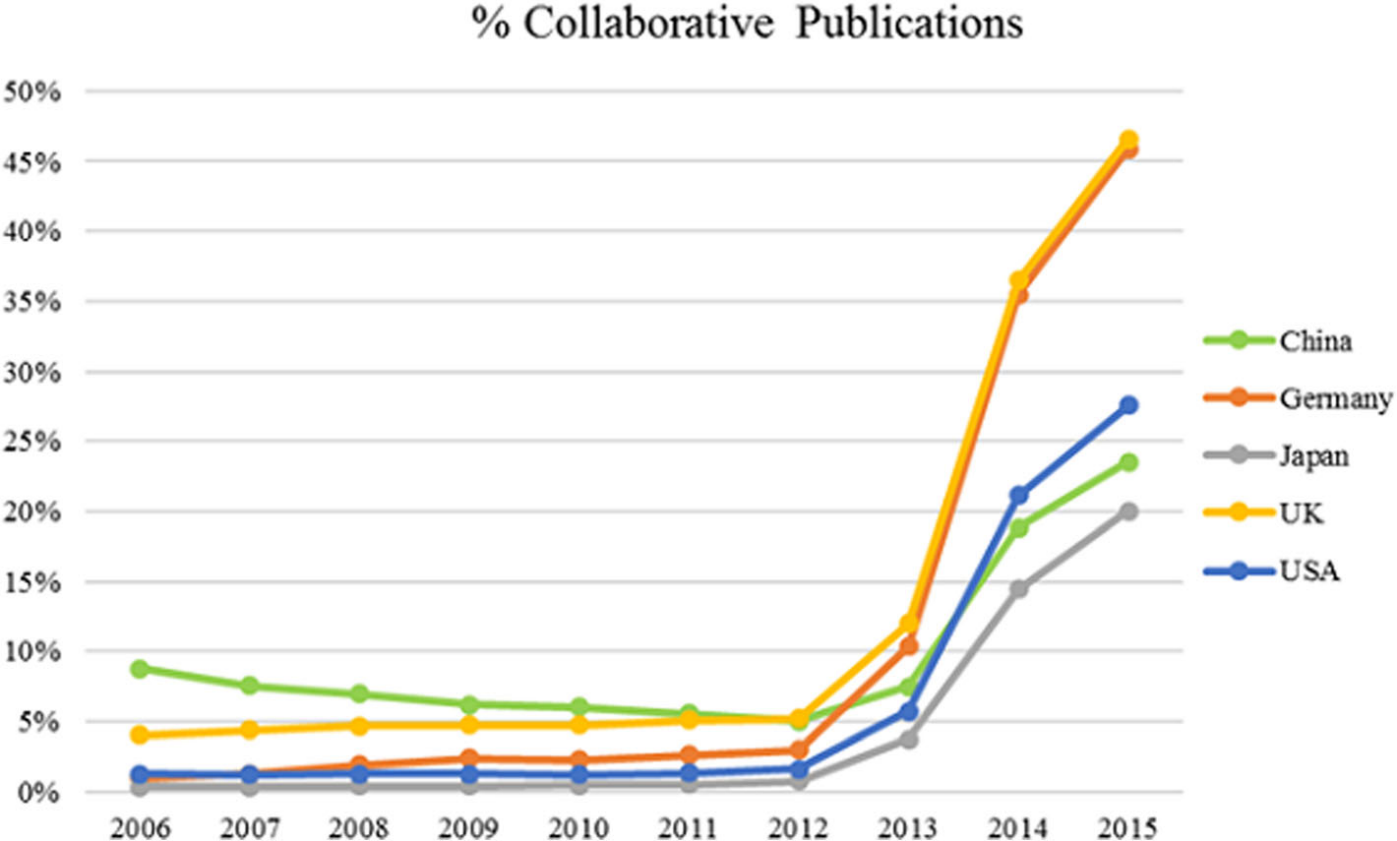
Percentage of collaborative publications from the five top publishing countries based on each country’s total publications, 2006–2015

Prior to October 2013, PubMed only indexed the first author’s affiliation. We wanted to determine how the change in policy of listing the affiliation of all authors made a difference, by comparing collaborative publication data for 10 years (2006 to 2015). Figure 3 illustrates the rapid increase in collaborative publications after 2013 as a result of this policy change. Although it can be attributed to the policy change, the increasing trend during the consecutive years may be real, stemming from increased collaborations between institutions internationally. Figure 3 also shows that even before 2013, there were already papers with multiple affiliations in PubMed entries, about 8% for China and 4% for the UK.

## Discussion

Recent reports of the rapid increase in meta-analysis publications from China prompted this study [4–7]. Our review seemed to confirm this trend. Although the majority of total journal articles were still published by researchers in the USA, data from 1995 to 2015 showed that China was almost equal in number to that of the USA in meta-analysis publications. China’s meta-analysis publications are at a steeper slope than any other country since it started in 2009 (Fig. 2) and has overtaken all countries in the top 10 category. In this review, we also found an increase in collaborative publishing among the top publishing countries.

Some reports cite evidence-based medicine as the stimulus for the rapid rise in the number of meta-analysis publications in China [4] and to the pressures of academia [6]. Ioannidis attributes the recent increase to contracting companies “operating in the domain of evidence synthesis” [5]. The tools for doing a meta-analysis and systematic reviews are widely accessible. However, this study did not show an increase in systematic reviews.

International collaboration improves the quality of research for all partners although it has some challenges as well [11]. The growth of collaborative research with China and other countries has been reported [11]. Publications in international journals from all countries have increased over the years as evidenced by total publications (Table 3 and Fig. 3). An even more positive development is the increased international collaborations between the top publishing countries (Fig. 3). In 2015 (Table 3), co-published papers between China and the USA accounted for 51.7% of China’s co-authored publications. Collaborations with other countries and regions were high as well (Hong Kong 14.4%, Taiwan 9.3%, Australia 7.3%, Japan 7.4%, Canada 6.5%, Germany 6%). In 2015, 27.6% of total US’ publications (34129) were collaborative publications. The most co-authored journal papers were with China, 19.8%; the UK, 16%; Canada, 12.2%; and Germany, 12%. The lack of data for all author affiliations before 2013 makes the trend of earlier international collaborative publications unclear. But from the first authors’ affiliations before 2013 (Fig. 3), we can infer that scientists working collaboratively have increased over the years.

Although we attempted to include all publications from the 235 countries with citations in PubMed, we may have missed some publications because some co-authors’ affiliations may not have been indexed in PubMed before 2013. Other reasons may include NLM indexing affiliations of authors only if provided by publishers in their XML submissions; association of authors with multiple institutions and not listing all affiliations; and discontinued editing of author affiliation field in MEDLINE/PubMed citations. In the past, NLM policy was to indicate the affiliation for the first author only and edited the field by adding “USA” (https://www.nlm.nih.gov/pubs/techbull/so13/brief/so13_author_affiliations.html). For example, many publications from the USA only list the states or even only the academic institution or the organization where they originated. After 2013, PubMed discontinued adding “USA” in the affiliation field. In our search algorithm, we added all the states in the USA to compensate for this. Some publications from China, Russia, or the Russian Federation that may have listed only their academic affiliations or cities would have missed their country affiliation. Unlike the authors in the USA, we cannot compensate by adding the names of all the states for China and Russia. However, a random search of over 200 articles from Russia and China all showed their countries in the affiliation field. It is unlikely, but this is still a possible limitation of the study.

Wilczynski and Haynes [12], analyzing the consistency and accuracy of several search strategies, found that using only single terms yielded low sensitivities—76.5% for “Review.pt” and 19% for “Meta analysis.pt.” In our search algorithms, we tried to include filters (Appendices 2, 3, and 4), similar to those used by Shojania and Bero [13] that yielded sensitivities between 93 and 97% and would identify publications that are systematic reviews and meta-analysis. However, there is no assurance that we have found all of these articles. This may be a limitation.

We found in this 20-year review of publications that only 30 countries (out of 235 total reviewed) are responsible for an average of 93.9% of all publications in PubMed. What might explain the few numbers of countries responsible for a majority of the world’s literature? It is likely a consequence of country priorities, lower levels of funding, and the high cost of doing research, although labor costs may be lower [14]. Biomedical research is often not a top priority in developing countries. However, although the research output of developing counties may be relatively low, many important medical and public health interventions were developed in developing countries, such as life-saving interventions like oral rehydration therapy for diarrhea and vitamin A to reduce infant mortality [15]. Moreover, collaborative research may be indexed as publications from funding agencies or collaborators in developed countries [11]. Other countries may also have fewer research and academic institutions. Developing countries can make important contributions to the medical literature, especially in the area of infectious diseases, and increasingly, non-communicable chronic diseases. Top research countries and research funding agencies may need to collaborate more with other countries. Some of this may be occurring through the internationalization of clinical trials that are now done worldwide and increased student and fellowship training [11].

We had also considered whether the rapid increase of publications might be explained by so-called predatory journals—low-quality journals with little or no peer review, which promise rapid publication but exist solely for profit [16]. However, we do not believe that these journals are responsible because this evaluation is based only in publications indexed in PubMed. Some data indicate that a few of these types of journals are indexed in PubMed. Moreover, Shen and Bjork also reported that “the problem of predatory open access seems highly contained to just a few countries, where the academic evaluation practices strongly favor international publication, but without further quality checks” [17].

## Conclusion

This study showed the continuing increase in research publications globally. The USA still leads the world in all publications and specific publication types except in meta-analysis where recently China now publishes more. Increasing collaborations and authorships among countries with the USA, China, and other top publishing countries is also increasing.

## Appendix 1

### Example of a filter for searching for country publications for Germany from 1995 to 2015

“Germany[affiliation] AND 2015/01/01 [PDAT] : 2015/12/31 [PDAT]”

## Appendix 2

### Filters for searching for systematic review and meta-analysis from 1995 to 2015 for China

systematic review [ti] AND (meta-analysis [pt] OR meta-analysis [ti] OR systematic review [tw] OR systematic review [tiab] OR meta synthesis [ti] OR meta-analy*[ti] OR cochrane database syst rev [ta] OR systematic literature review [ti] OR pooling project [tw] OR umbrella review [tw] )AND “1995/01/01”[PDAT] : “2015/12/31”[PDAT] AND China[affiliation]

## Appendix 3

### Filters to search for systematic reviews only from 1995 to 2015 replace China with name of country

(systematic review [tw] OR systematic review [tiab] OR meta synthesis [ti] OR cochrane database syst rev [ta] OR systematic literature review [ti] OR pooling project [tw] OR umbrella review [tw] )AND “1995/01/01”[PDAT] : “2015/12/31”[PDAT] AND China[affiliation]

## Appendix 4

### Filters to search for meta-analysis only from 1995 to 2015 for China

(meta-analysis [pt] OR meta-analysis [ti] OR meta synthesis [ti] OR meta-analy*[ti] ) AND “1995/01/01”[PDAT] : “2015/12/31”[PDAT] AND China[affiliation]

## Appendix 5

### Filters to search for meta-analysis only the Mexico (country) only, not New Mexico state

(meta-analysis [pt] OR meta-analysis [ti] OR meta synthesis [ti] OR meta-analy*[ti] ) AND “1995/01/01”[PDAT] : “2015/12/31”[PDAT] AND mexico[affiliation] NOT new mexico[affiliation]
